# Primary and Metastatic Pancreatic Ewing Sarcomas: A Case Report and Review of the Literature

**DOI:** 10.3390/diagnostics14232694

**Published:** 2024-11-29

**Authors:** Nektarios I. Koufopoulos, Menelaos G. Samaras, Christakis Kotanidis, Konstantinos Skarentzos, Abraham Pouliakis, Ioannis Boutas, Adamantia Kontogeorgi, Magda Zanelli, Andrea Palicelli, Maurizio Zizzo, Giuseppe Broggi, Rosario Caltabiano, Anastasios I. Kyriazoglou, Dimitrios Goutas

**Affiliations:** 1 Second Department of Pathology, Medical School, National and Kapodistrian University of Athens, Attikon University Hospital, 15772 Athens, Greece; mgsamaras@med.uoa.gr (M.G.S.); xrkotan@gmail.com (C.K.); k.skarentzos@gmail.com (K.S.); apou1967@gmail.com (A.P.); dimgoutas@med.uoa.gr (D.G.); 2 Breast Unit, Rea Maternity Hospital, P. Faliro, 17564 Athens, Greece; drboutas@gmail.com; 3 Third Department of Obstetrics and Gynecology, Medical School, National and Kapodistrian University of Athens, Attikon University Hospital, 15772 Athens, Greece; ad.kontogewrgi@gmail.com; 4 Pathology Unit, Azienda USL-IRCCS di Reggio Emilia, 42123 Reggio Emilia, Italy; magda.zanelli@ausl.re.it; 5 Surgical Oncology Unit, Azienda USL-IRCCS di Reggio Emilia, 42122 Reggio Emilia, Italy; maurizio.zizzo@ausl.re.it; 6 Clinical and Experimental Medicine PhD Program, University of Modena and Reggio Emilia, 41121 Modena, Italy; 7 Department of Medical and Surgical Sciences and Advanced Technologies “G.F. Ingrassia”, 95123 Catania, Italy; giuseppe.broggi@phd.unict.it (G.B.); rosario.caltabiano@unict.it (R.C.); 8 Second Propaedeutic Department of Medicine, Oncology Unit, National and Kapodistrian University of Athens, Attikon University Hospital, 15772 Athens, Greece; tassoskyr@gmail.com

**Keywords:** Ewing sarcoma, primitive neuroectodermal tumor, pancreas, metastasis

## Abstract

Ewing sarcomas are rare tumors arising mainly in the bones and the surrounding soft tissues. Primary extraosseous Ewing sarcomas have also been described in several other organs and locations other than bones, including the pancreas. These tumors have well-defined histological, immunohistochemical, and molecular characteristics. In this manuscript, we present a case of primary Ewing sarcoma of the pancreas in a 29-year-old patient, and we systematically review the literature on both primary and metastatic Ewing sarcomas of the pancreas, describing their clinicopathological characteristics. We also discuss the differential diagnosis and the treatment of this rare entity.

## 1. Introduction

Primary pancreatic carcinoma ranks as the third most common cause of cancer-related death in the U.S. [1]. Metastases to the pancreas are far less common. There are reports of several different malignancies, including melanoma, breast, lung, gastrointestinal tract, and renal carcinomas, as well as lymphomas [2]. It is very difficult to calculate the true incidence of pancreatic metastases. They range from 1.6% to 39% during autopsies of patients with cancer, depending on the primary tumor [2]. In most instances, patients with pancreatic metastases have widespread disease with a multitude of other metastatic sites [2].

Ewing sarcoma (ES) is a rare tumor involving the bones or soft tissue surrounding the bone. It is the second most common primary malignant bone tumor. Its peak incidence is in the second decade of life. Almost 80% of patients are younger than 20 years of age and it is uncommon in patients older than 30 years [3]. It is a poorly differentiated and aggressive small-blue-round-cell neoplasm of neuroectodermal origin that affects children and young adolescents. It most commonly affects Caucasians and, less commonly, Asians and African Americans [4]. It was first described in 1921 by James Ewing as a diffuse endothelioma of the bone. Tefft first described the extraosseous form of ESs in 1969 [5]. The ES family of tumors includes entities such as classical ES, extraosseous ES, peripheral primitive neuroectodermal tumor (PNET), and Askin tumor of the chest wall. These tumors share common morphological features, being composed of small blue round cells with extensive areas of necrosis but viable tumors usually retained around blood vessels, an immunohistochemical profile expressing the MIC2-protein (CD99), and cytogenetics displaying the same chromosomal translocation t (11; 22) (q24; q12) in about 85% of the cases [5]. These sarcomas are prone to metastatic involvement. Lung, pleura, and other bones are the most common metastatic sites. Several other metastatic sites have been described. However, pancreatic metastasis is very rare, with few reported cases in the English literature.

On the other hand, primary extraosseous ES/PNETs have been described in a variety of organs, including the kidney, urinary bladder, ureter, prostate, penis, seminal vesicle, testis, small bowel, rectum, liver, gall bladder, maxillary sinus, trachea, lung, parotid gland, vulva, vagina, ovary, uterine cervix, uterus, and breast [3].

A primary extraosseous ES/PNET of the pancreas is a very rare tumor, with around 50 cases reported in the English literature.

Regarding the prognosis of these tumors, the presence of metastatic disease is the most important prognostic factor [3].

Also, another favorable prognostic factor is the complete pathologic response to neoadjuvant chemotherapy. In contrast, the presence of early relapse and its occurrence in the trunk and pelvis predicts an unfavorable outcome [6].

In this manuscript, we present a case of primary ES/PNETs of the pancreas and review the literature on primary and metastatic ES/PNETs of the pancreas. We also discuss the differential diagnosis and treatment strategy of these tumors. Finally, we performed a statistical analysis based on the collected data from individual patients to evaluate the potential role of metastatic disease on patient survival and disease recurrence.

## 2. Materials and Methods

The patient provided written informed consent to participate in this study. The case report (involving a human participant) was reviewed and approved by the Institutional Review Board of Attikon University Hospital (ΕΒΔ210/27-03-2023).

We performed a systematic review of the literature according to the PRISMA (“Preferred Reporting Items for Systematic Reviews and Meta-Analyses”) guidelines (http://www.prismastatement.org/; accessed on 15 July 2024).

Our retrospective observational study search was conducted through the PICO process:

- Population: Men or women with a diagnosis of primary or metastatic ESs of the pancreas;
- Intervention: Surgical treatment of the primary or metastatic ES;
- Comparison: None;
- Outcome: Patients’ treatment and follow-up.

We searched for Ewing sarcomas involving the pancreas on PubMed (all fields; 116 results; https://pubmed.ncbi.nlm.nih.gov, accessed on 15 July 2024), Scopus (title/abstract/keywords; 528 results; https://www.scopus.com/home, accessed on 15 July 2024), and Web of Science (all fields; 118 results; https://login.webofknowledge.com, accessed on 15 July 2024) using the terms ((“Ewing”) AND (“Sarcoma”) AND (“pancreas” OR “pancreatic”)). We did not set any additional limitations while performing the search. We applied the following criteria:

- Eligibility/inclusion criteria
  (1) Study design: We only included original studies and case reports describing cases of primary pancreatic ES and ES metastatic to the pancreas.
  (2) Population: Studies involving patients diagnosed with ES that provided adequate surgical and/or oncological information.
  (3) Intervention or exposure: We included studies that examined any treatment or intervention for ES, including surgery, chemotherapy, radiation therapy, or targeted therapies.
  (4) Outcome: We included studies that reported on the presence or absence of disease relapse as an outcome measure.
  (5) Language: The included studies were written in the English language.
- Exclusion criteria
  (1) Review articles and editorials: We excluded narrative or systematic reviews, meta-analyses, opinion pieces, and other articles that did not present original research findings.
  (2) Insufficient information: We excluded cases with insufficient or too much aggregated data.
  (3) Language: We excluded manuscripts in languages other than English.
  (4) Uncertain diagnosis: Cases with an uncertain/doubtful diagnosis were excluded.

**Figure 1:**
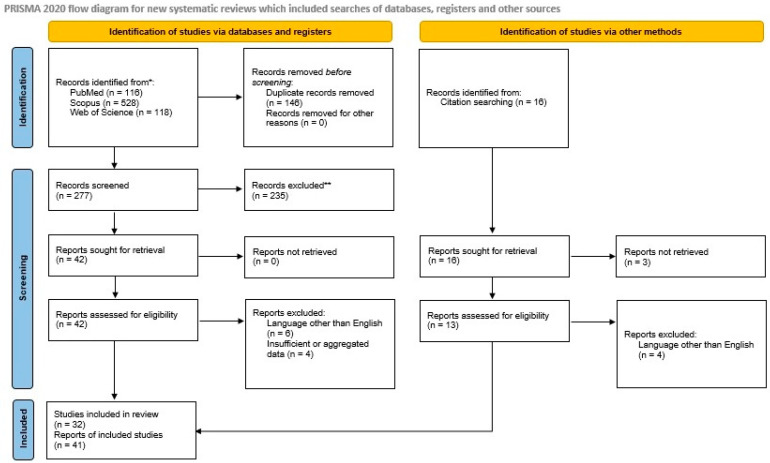
The PRISMA 2020 flowchart shows the search strategy, excluded studies, and, finally, the included primary and metastatic pancreatic Ewing sarcoma reports.

We included all primary articles and case reports in the English language describing primary and metastatic ESs of the pancreas. We excluded abstracts from medical conferences, previous review articles, and articles describing cases with unclear diagnoses and too much missing or aggregated data. Two authors [NK and MGS] reviewed the literature and collected data. Discrepancies were corrected in consensus. After applying inclusion and exclusion criteria, 33 manuscripts describing 51 primary [7,8,9,10,11,12,13,14,15,16,17,18,19,20,21,22,23,24,25,26,27,28,29,30,31,32,33,34,35,36,37,38,39] cases and 8 manuscripts describing 8 metastatic ES/PNET cases to the pancreas [40,41,42,43,44,45,46,47] remained for data extraction. A PRISMA flowchart with a summary of search results is shown in Figure 1.

Statistical analysis was performed within the environment of the R language (version 4.4.0). Continuous variables were expressed as mean ± standard deviation when normally distributed and as a median minimum with a quartile 1 to quartile 3 range and minimum and maximum values; the categorical data are presented as frequency and the relevant percentage. Paired t-tests or Mann–Whitney U test were conducted to compare changes between groups according to normality conditions as this was evaluated by the Shapiro–Wilk test. Survival was analyzed with Kaplan–Meier estimates and log-rank tests. The significance level was set to 0.05, and tests were two-sided when appropriate.

## 3. Results and Discussion

### 3.1. Detailed Case Description

A 29-year-old male patient was admitted to the hospital due to a history of abdominal pain for two months and jaundice. Past medical history as well as family history were unremarkable. An abdominal ultrasound revealed a heterogeneous mass located in the head of the pancreas. A computed tomography (CT) scan showed a mixed-density lesion of the head of the pancreas with a size of about 40 mm with high suspicion of pancreatic cancer. Serum markers were within normal limits. A CT-guided fine-needle aspiration biopsy was performed.

**Figure 2:**
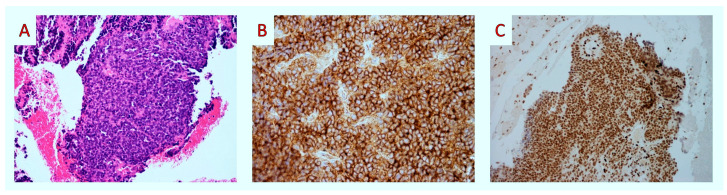
Fine-needle aspiration biopsy: histopathologic findings. (**A**) Upon medium-power examination, the tumor was composed of uniform small blue round cells (H&E × 100). (**B**,**C**) After immunohistochemistry, tumor cells showed membranous staining for CD99 (CD99 × 100) and nuclear staining for FLI-1 (FLI-1 × 40).

Histologically, tumor cells were small, round, and discohesive and had irregular nuclear membranes with occasional cleaving (Figure 2A). There was no finely granular “salt-and-pepper” chromatin texture consistent with neuroendocrine neoplasm. Nucleoli were absent. There were several mitotic figures per 10 high-power fields. There was no evidence of glandular differentiation, Homer-Wright rosettes, or organoid configuration. Also, distinct nuclear molding was not present. An extensive immunohistochemical panel was applied. Membranous CD99 (mouse monoclonal BSB-9, Bio SB) (Figure 2B) and nuclear FLI1 (mouse monoclonal G146-222, Medac Gmbh) (Figure 2C) were positivity was noted. In contrast, CKAE1/AE3 (mouse monoclonal AE1/AE3, Dako), CK8/18 (mouse monoclonal 5D3, Thermoscientific), Chromogranin (mouse monoclonal DAK-A3, Dako), Synaptophysin (mouse monoclonal DAK-Synap, Dako), CD56 (rabbit monoclonal RCD-56, Zytomed), SMA (mouse monoclonal 1A4, Dako), and Desmin (mouse monoclonal D33, Zytomed) were negative. Ki67 (rabbit monoclonal EP1, Dako) stained 40–50% of tumor cells. Based on the above results, we made the diagnosis of malignant neoplasm, which was morphologically and immunohistochemically consistent with an ES. Molecular testing revealed an EWSR1-FLI1 gene fusion, considered pathognomonic for diagnosing ESs.

The patient received six cycles of neoadjuvant therapy consisting of CAV (vincristine, adriamycin, and cyclophosphamide) alternating with IE (ifosfamide and etoposide). The patient tolerated the treatment well. After completion of neoadjuvant therapy, a pancreatoduodenectomy was performed. Histological evaluation of the specimen showed extensive necrosis with no viable tumor cells. The patient’s recovery was uneventful. He received five additional cycles of CAV/IE. Two and half years after the diagnosis, imaging studies revealed metastasis to the femur and lower lobe of the right lung. A lobectomy was performed, and the patient received irinotecan and temozolomide. After three cycles, imaging studies showed disease progression. The patient died a few weeks later, 37 months after the initial diagnosis.

### 3.2. Primary Pancreatic Ewing Sarcoma

#### 3.2.1. Demographic and Clinicopathological Features

Primary pancreatic ES is a rare entity. Our review found 33 manuscripts describing 51 cases of primary pancreatic ES/PNET. Out of 50 patients, 28/50 (56%) [8,9,10,12,13,14,19,22,23,27,28,29,30,34,37,38,39] were male, and 22/50 (44%) [7,9,11,15,16,17,18,21,24,25,26,30,31,32,33,35,36] were female. In one case, the patient’s gender was not mentioned [20]. The mean patient age was 26 years (range 2–78 years). The mean tumor size was 80.4 mm (range 30–200 mm). The specific location of the tumor was mentioned in 49/51 (96%) [7,8,9,10,11,12,14,15,16,17,18,19,20,21,22,23,24,25,26,27,28,29,30,31,32,33,34,35,36,37,38,39] cases. The head of the pancreas was the most common location in 26/49 (53%) [7,8,9,14,16,20,22,23,28,30,32] cases, followed by the body and tail in 10/49 (20.4%) [15,17,19,26,28,30,31,36,37,39] cases, the body in 5/49 (10.2%) [11,21,27,30] cases, the tail in 2/49 (4.1%) [12,34] cases, the head and uncinate process in 2/49 (4.1%) [25,33] cases, and the head and tail [10], the uncinate process [18], the head and body [24], and the body and neck [34] in 1/49 (2%) cases each. Symptoms were mentioned in 47/51 (92.1%) [7,9,10,11,12,13,14,15,16,17,18,19,20,21,22,23,24,25,26,27,28,29,30,31,32,33,34,35,36,37,38,39] patients. The most common symptom was abdominal or epigastric pain in 35/47 (74.5%) [9,12,14,16,17,18,19,20,22,23,24,26,27,28,29,30,31,34,36,37,39] cases followed by jaundice in 14/47 (29.8%) [9,13,24,25,28,30] patients, loss of weight in 5/47 (10.6%) [18,27,29,33,39] cases, loss of appetite in 5/47 (10.6%) patients, abdominal mass in 4/47 (8.5%) [23,29,36,38] patients, nausea in 3/47 (6.4%) [19,31,36] patients, vomiting in 3/47 (6.4%) [12,31,35] patients, and fatigue in 2/47 (4.2%) [7,14] patients. A multitude of other symptoms were also present in the patients. The demographic, clinicopathological, and treatment features of the cases are shown in Table 1.

#### 3.2.2. Imaging Findings

Imaging findings are not specific for this type of tumor. On pre-contrast CT images, the tumors are isodense with regions of necrosis. Contrast-enhanced CT images display heterogeneity with mild to medium enhancement and patchy intratumor unenhanced areas [48]. ESs appear typically isointense on T1-weighted images and display variable isointensity or hyperintensity on T2-weighted images on MRIs [49]. Since these findings are similar to pancreatic carcinoma, the accurate diagnosis of ESs of the pancreas depends on the results of the histopathologic examination.

#### 3.2.3. Histological Findings and Differential Diagnosis

The diagnosis of ES can be particularly challenging. Clinical, radiological, pathological, and cytogenetic data can provide valuable diagnostic information.

Grossly, the tumor has a grayish-white color. Microscopically, primary extraskeletal Ewing sarcomas are characterized by small (1–2× size of lymphocytes), uniform, monotonous, round cells with finely stippled chromatin, inconspicuous nucleoli, scant cytoplasm, and indistinct cytoplasmic membranes. Tumor cells are arranged in a sheet-like growth pattern in islands separated by dense fibrous tissue. Tumor necrosis can be seen in several cases. Sometimes, there is neuroectodermal differentiation evidentiated by the presence of Homer-Wright pseudorosettes [50]. One of the cases showed adamantinoma-like Ewing sarcoma morphology [37], which is characterized by nests of basaloid cells, peripheral palisading and cording, prominent myxoid, fibromyxoid or hyalinized stroma, focal keratin pearl formation, and high-grade features with minimal pleomorphism [51]. Immunohistochemically, tumor cells show strong and diffuse membranous expression for CD99 [52], positivity for NKX2.2 [53], and Vimentin in 80–90% of cases. FLI1 shows nuclear staining in around 90% of cases with EWSR1-FLI1 fusion [54,55]. ERG displays nuclear staining in cases with EWSR1-ERG fusion [56]. Cases with adamantinoma-like morphology show diffuse positivity for pan-cytokeratin, CK5/6, p63, and p40. ES/PNETs may sometimes express neuroendocrine markers such as Synaptophysin and/or CD56 [3]. The histological differential diagnosis of ES/PNETs includes small-cell neuroendocrine carcinoma, a more typical finding in the pancreas [57]. ES and small-cell carcinoma have a similar morphology, composed of small round cells with minimal cytoplasm [58]. In imaging studies, these entities have similar findings with irregular borders and heterogeneous enhancement [59]. It is important to differentiate between these tumors since the treatment is distinct. Another pancreatic tumor that enters the differential diagnosis is solid pseudopapillary neoplasm. Upon imaging, it is usually well defined and heterogeneous with cysts [45]. Other differential diagnoses considered are desmoplastic small-round-cell tumors and pancreatoblastomas since they have similar morphological findings with the ES family of tumors (small monomorphic round cells with small nuclei and scant cytoplasm). In desmoplastic small-round-cell tumors, one can notice desmoplasia, while immunohistochemically, tumor cells are positive for cytokeratin and Desmin [60]. Pancreatoblastomas display, microscopically, cells with acinar-like differentiation and the formation of small squamoid nests [61]. In most cases, immunohistochemical analysis provides a diagnostic solution. An ES is usually positive for CD99 and FLI-1 in contrast to small-cell carcinoma and solid pseudopapillary neoplasm. Markers of epithelial (AE1/AE3, EMA, CK8/18, and CK7) and neuroendocrine differentiation (NSE, Chromogranin A, Synaptophysin, and CD56) may display variable results in ESs [3]. In difficult cases, molecular analysis by fluorescence in situ hybridization (FISH) or reverse transcription–polymerase chain reaction (rt-PCR) of the EWSR-FLI1 fusion gene, which is specific to ESs, can help confirm the diagnosis.

#### 3.2.4. Molecular Studies

In ES/PNETs, the result of the EWS-FLI1 gene fusion is the karyotype of t(11;22) (q24;q12), and the EWS-ERG gene fusion results in t(21;22) (q22;q12), which account for 85% and 10%, respectively [62,63,64]. On rare occasions, an ES shows fusions of EWS to other ETS-family genes (ETV1, ETV4, and FEV) or similar fusions of the EWS-related gene FUS (FUS-ERG or FUS-FEV) [65,66].

Molecular studies have been performed in the majority of pancreatic ESs. Most cases displayed the t(11;22) (q24;q12) translocation while fewer displayed the 22q12 rearrangement.

#### 3.2.5. Treatment

Surgical treatment data were reported in all cases. In 24/51 (47%) cases, treatment consisted of a Whipple procedure [7,8,10,13,14,16,22,23,25,30,33,38] or distal pancreatectomy in 5/51 (9.8%) [11,15,17,19,26,31] cases, en bloc resection of pancreas and kidney in 2/51 (3.9%) [30], central pancreatectomy in 1/51 (1.9%) [35], gastropancreatoduodenectomy in 1/51 (1.9%) [24], left pancreatectomy in 1/51 (1.9%) [12], and total mass excision in 1/51 (1.9%) [34]. Biopsies were performed in 4/51 (9.3%) [9,21] cases, and open laparotomy and surgical biopsies were taken in 2/43 (4.6%) [20,27] cases. Additionally, in some cases, other procedures such as Roux-en-Y choledochojejunostomy [28,29], splenectomy [12,15,17,19,26,31,39], partial gastric resection [12], nephrectomy [34], adrenalectomy [34,37], and colectomy [31,38] were performed.

Information regarding adjuvant treatment was provided in 38/51 (74.5%) [7,8,9,10,11,12,13,14,15,16,17,18,19,20,21,23,24,25,26,28,29,30,31,32,34,35,36,37,38] cases. Chemotherapy either in the adjuvant or neoadjuvant setting was offered in 30/38 (78.9%) [9,11,12,13,14,15,16,17,18,19,20,21,23,25,26,29,30,31,34,35,37,38] cases and radiotherapy in 5/33 (15.1%) [13,17,18,25,34] cases. In three cases, patients or their families refused the proposed chemotherapy [7,8,10]. The most common regimen used consisted of vincristine, adriamycin, and cyclophosphamide alternating with ifosfamide and etoposide, while the second most common regimen consisted of vincristine, adriamycin, and cyclophosphamide administered in eight and six cases, respectively. Among ten cases with recurrence, six received additional chemotherapy, sometimes combined with surgery [8,10,12,17,18,30], two received other treatment (surgical for the first and radioablation for the second case) [13,27], and three patients received no further treatment [7,28,31]. ES/PNETs are famous for displaying extremely malignant behavior with frequent relapse and metastasis. The treatment of ESs consists of surgery followed by chemotherapy.

A five-drug regimen (vincristine, adriamycin, cyclophosphamide, ifosfamide, and etoposide) has been established as the gold standard for treating ES/PNETs [64]. Radiation therapy can be used with some therapeutic efficacy in patients with residual disease [43].

#### 3.2.6. Outcome

Follow-up information was available in 38/51 (77.5%) [7,8,9,10,11,12,13,15,16,17,18,20,23,24,26,28,29,30,31,32,34,35,36,37,38,39] cases. Briefly, 18/38 (47.4%) patients were alive without evidence of disease [9,11,12,15,23,24,26,29,30,34,35,37,38], 6/38 (15.8%) were alive with disease [8,9,13,30], and 12/38 (31.6%) died of disease [7,9,10,17,18,30,32,36] in a timeline ranging from 1 to 120 months. In two out of thirty-eight (5.2%) cases, patients died of other causes [16,28], one of them due to postoperative complications and the second due to severe infection and multiple organ failure. A small number of studies have claimed that an extraskeletal ES has a more favorable prognosis compared to its skeletal counterpart [67,68]. Tural et al. have reported that tumor size equal to or exceeding 8 cm is a significant predictor of worse overall survival [69].

### 3.3. Ewing Sarcoma Metastasis to the Pancreas

#### 3.3.1. Demographic and Clinicopathological Features

Metastatic disease of the pancreas is rare. It accounts for 2% of all pancreatic cancers [70]. The primary tumors that most frequently provide pancreatic metastasis are lung cancer, renal cell carcinoma, breast cancer, and melanoma [71]. However, it should be noted that pancreatic metastasis is a good prognostic factor for renal cell carcinoma, with excellent response to treatment with tyrosine kinase inhibition [72].

The prognosis of metastasis to the pancreas is poor. In soft tissue sarcoma, the role of surgical treatment is not clear. Reports of prolonged survival after removal of isolated metastatic foci have been published for different neoplasms, including carcinomas [73,74,75] and soft tissue sarcomas [76].

Metastatic ESs to the pancreas have been reported even less frequently than primary ES/PNETs of the pancreas. Our review found eight articles describing eight ES cases with metastasis to the pancreas. Gender was reported in all cases, with 7/8 (87.5%) [40,41,42,43,45,46,47] patients being male and 1/8 (12.5%) [44] being female. The individual patient’s age was reported in all cases. The mean age was 23.2 years (range 13–37 years). The mean tumor size of the metastatic focus was 29.7 mm (range 21–40 mm). The most common symptoms are not specific, including abdominal or epigastric pain in 3/8 (37.5%) [42,44,45] patients and vomiting in 2/8 (25%) [44,45] patients. Other less common symptoms include nausea and jaundice [45]. These symptoms are similar to those of primary pancreatic ES/PNETs. The summary of the demographic, clinicopathological, and treatment features of these cases is displayed in Table 2.

#### 3.3.2. Imaging Findings

The imaging features of metastatic ESs to the pancreas are not pathognomonic. They show solid and cystic components [42], a hypodense mass [45], a heterogeneously enhancing lesion [47] on computed tomography scans, or a hypoechoic mass on endoscopic ultrasounds [43].

#### 3.3.3. Histological Findings

On microscopic examination, the histologic appearance of metastatic ES/PNETs is identical to that of the primary one, which consists of small, round neoplastic cells. The same is true for immunohistochemistry, which displays positivity for CD99, NKX2.2, either FLI-1 or ERG, and sometimes for Chromogranin, Synaptophysin, and CD56.

#### 3.3.4. Molecular Studies

In two cases, the presence of EWSR1-FLI1 rearrangement was mentioned.

#### 3.3.5. Treatment

There was information regarding surgical treatment in 7/8 (87.5%) [40,41,42,44,45,46,47] cases. In 5/7 (71.4%) [40,44,45,46,47] patients, surgical treatment was not performed. In 1/7 (14.3%) [42], the patient underwent left lung lobectomy and lymph node dissection, and in 1/7 (14.2%) [41], an open surgical biopsy was performed.

Detailed information regarding adjuvant therapy was provided for 7/8 (87.5%) [40,41,42,43,45,46,47] patients. Chemotherapy was administered in 6/7 (85.7%) [40,41,43,45,46,47] patients; 2 received the VAC-IE regimen and 1 received VAC. In one case, prednisone, vincristine, and cyclophosphamide (for lymphoma diagnosis) were administered because the neoplasm was misdiagnosed as a lymphoma [40]. In another case, the patient received cisplatin and etoposide due to small-cell lung carcinoma misdiagnosis, which was later changed to a VAC regimen [45]. Radiotherapy was provided in 3/7 (42.8%) [41,45,46] patients. In cases of recurrence, patients received adjuvant chemotherapy and, in some cases, surgical treatment and radiotherapy. In 1/7 (14.3%) [42] cases, the patient refused adjuvant chemotherapy. It should be noted that according to the REECUR trial, the preferred regimen for metastatic or recurrent ESs is ifosfamide [77]. On the other hand, trials reported with tyrosine kinase inhibitors such as regorafenib or cabozantinib in metastatic/recurrent ESs do not describe the activity of these regimens in patients with pancreatic metastases [78,79].

#### 3.3.6. Outcome

Follow-up information was available for 6/8 (75%) [40,41,42,43,45,46] patients. Follow-up time ranged from 5 to 402 months (mean 93). In 4/6 (66.7%) [42,43,45,46] cases, patients were alive with the disease, and 2/6 (33.3%) [40,41] succumbed to the disease.

### 3.4. Patient Survival, Disease Recurrence, and Comparisons Between Survivors and Non-Survivors and Patients with Recurrence and Non-Recurrence

**Figure 3:**
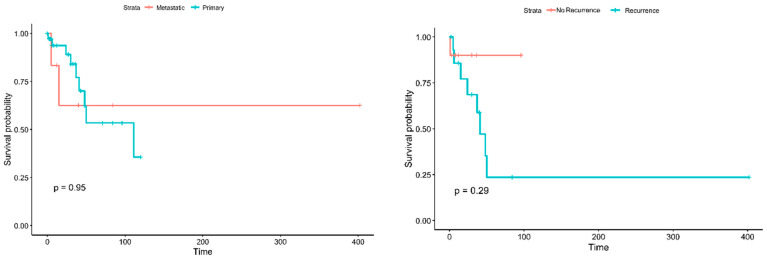
Kaplan–Meier curves for the survival of patients with metastatic vs. primary tumors (**left**) and for the patients with and without recurrence (**right**).

As the collected data had details for each patient, it was feasible to perform various inferential statistics to evaluate possible differences related to diseases, patients, or other characteristics. Differences in the survival of patients with metastatic disease from patients with primary tumors were not possible to be confirmed (*p* = 0.95) (see Figure 3) nor were the differences between the groups of patients with and without recurrence (*p* = 0.29). Notably, there is a trend for better survival of the patients without recurrence; however, larger data sets are required for robust statistical confirmation.

A detailed analysis among patients with metastatic (N = 8) and with primary ESs (N = 52) showed a marginal difference (*p* = 0.0857) for abdominal pain as a symptom. More specifically, 63% of the patients with primary tumors had abdominal pain, while only 25% of the patients with metastatic disease displayed this symptom. Furthermore, differences were found (*p* = 0.0183) in the patient’s status (as reported in the studies), with 35% of the patients with primary tumors being reported as alive without evidence of disease, while none of the patients with metastatic disease was reported to be cured (see Supplementary Table S1 for details). Additional analysis of the patients with recurrent disease (N = 19) and those without (N = 13) indicates that the major difference lies in the life status during the study, as 47.4% of the patients with recurrence were deceased due to the disease while only 15.4% of the patients without recurrence succumbed to the disease (see Supplementary Table S2 for more details).

## 4. Conclusions

In conclusion, we have presented a new case of primary ESs of the pancreas and reviewed the literature on primary and metastatic ESs of the pancreas. Both represent rare entities, with few cases reported in the English literature. Further studies of these tumors with long-term follow-ups need to be reported to fully understand their behavior.

## Figures and Tables

**Table 1:** Clinicopathological and treatment data of primary ES/PNETs of the pancreas.

Authors	Year	Age	Gender	Clinical Presentation	Site	Tumor Size (cm)	Surgery	CHT/RT	Recurrence	Interval to Recurrence (Months)	Second-Line Therapy	Outcome (mo)
Bulchmann et al. [7]	2000	6	F	Fatigue, paleness, dizziness	Head of the pancreas, duodenal invasion	5.4	Whipple	None (family refusal)	Yes	6	None	6 DOD
O’Sullivan et al. [8]	2001	20	M	NM	Head of the pancreas	3.5	Whipple	None (patient refusal)	Yes	30	Left upper lobe lesion excision + CHT	30 AWD
Movahedi-Lankarani et al. [9]	2002	17	M	Abdominal pain, jaundice	Head of the pancreas	9	Whipple	VAC	NM	NM	NM	33 ANED
Movahedi-Lankarani et al. [9]	2002	20	M	Abdominal pain, jaundice	Head of the pancreas	3.5	Whipple	No	NM	NM	NM	27 AWD
Movahedi-Lankarani et al. [9]	2002	21	F	Abdominal pain	Head of the pancreas	NM	Whipple	NM	NM	NM	NM	0 DOC
Movahedi-Lankarani et al. [9]	2002	25	F	Abdominal pain	Head of the pancreas	NM	Biopsy	NM	NM	NM	NM	NM
Movahedi-Lankarani et al. [9]	2002	25	F	Abdominal pain, jaundice	Head of the pancreas	8	Biopsy	NM	NM	NM	NM	NM
Movahedi-Lankarani et al. [9]	2002	13	M	Abdominal pain	Head of the pancreas	6	Biopsy	NM	NM	NM	NM	43 ANED
Movahedi-Lankarani et al. [9]	2002	6	M	Abdominal pain, jaundice	Head of the pancreas	3.5	Whipple	VAC	Yes	48	NM	48 DOD
Perek et al. [10]	2003	31	M	Malaise, fever	Head and tail of the pancreas	10	Whipple	None (patient refusal)	Yes	4, 24, 36	Mass excision en bloc with right kidney + CHT/lung wedge resection + CHT/CHT + palliative care	50 DOD
Schutte and Knight [11]	2006	2	F	Pubic hair, breast bud development, vaginal bleeding	Body of the pancreas	6	Distal pancreatectomy	VAC–cisplatin and VP-16	NM		NM	12 ANED
Welsch et al. [12]	2006	33	M	Abdominal pain, vomiting	Tail of the pancreas	18	Partial gastric resection (Billroth II) + left pancreatic resection + splenectomy	6 × vincristine, ifosfamide, doxorubicin and etoposide; 1 × vincristine, actinomycin D (ifosfamide), followed by CHT with melphalan and etoposide and autologous stem cell transplantation	Yes	1	Initial adjuvant chemotherapy plan	12 ANED
Doi et al. [13]	2009	37	M	Jaundice	Pancreas, hepatic metastases	6	Whipple	VAC-IE + RT	Yes	12	Radio ablation of hepatic metastases	12 AWD
Maxwell et al. [14]	2011	11	M	Abdominal pain, fatigue	Head of the pancreas, post-ampullary ulcer	9.8	Whipple	Neoadjuvant VAC-IE	NM	NM	NM	NM
Bose et al. [15]	2012	31	F	Pancreatitis	The body and tail of the pancreas	3	Distal pancreatectomy, splenectomy, cholecystectomy	Adjuvant VAC-IE	No	-		12 ANED
Dias et al. [16]	2013	25	F	Abdominal pain	Head of the pancreas	4	Whipple	1× vincristine, actinomycin D, and ifosfamide, followed by 2 × VAC	No	-	-	8 DOC
Jayant et al. [17]	2013	20	F	Abdominal pain	The body and tail of the pancreas	11	Distal pancreatectomy, splenectomy	Adjuvant VAC + RT	Yes	24	CHT + RT	24 DOD
Mao et al. [18]	2013	13	F	Abdominal pain, polyuria, polydipsia, anorexia, weight loss	Uncinate process of the pancreas, colon, and greater omentum	3.5	Uncinate process resection + partial transverse colon resection	Adjuvant VAC + RT	Yes (4)	9, 12, 36, 40	R0 liver mass resection + ifosfamide, mesna, epirubicin, imidazole, R0 peritoneal mass resection + right hemicolectomy, RT	41 DOD
Reilly et al. [19]	2013	23	M	Abdominal pain, nausea	The body and tail of the pancreas	5.8	Distal pancreatectomy, splenectomy	Adjuvant therapy	NM	NM	NM	NM
Changal et al. [20].	2014	60	NM	Epigastric pain	Head of the pancreas	4	Surgical biopsy	Vincristine, ifosfamide, doxorubicin, and etoposide	NM	NM	NM	3 AWD
Kim et al. [21]	2014	58	F	Incidental finding	Body of the pancreas	NM	Biopsy	Therapeutic CHT	NM	NM	NM	NM
Nishizawa et al. [22]	2015	22	M	Abdominal pain	Head of the pancreas	8.5	Whipple	Adjuvant therapy	NM	NM		NM
Kumar et al. [23]	2015	22	M	Abdominal pain, palpable abdominal lump	Head of the pancreas	20	Whipple	Adjuvant CHT	No	NA	NA	3 ANED
Teixeira et al. [24]	2015	28	F	Epigastric pain, pruritus, jaundice, choluria, acholia	Head and body of the pancreas	11.5	Gastroduodenopancreatectomy	No adjuvant treatment	No		No	36 ANED
Golhar et al. [25]	2017	17	F	Jaundice, itching, loss of appetite, pale-colored stools, hematemesis	Head and uncinate process of the pancreas	7.5	Whipple	Adjuvant VAC + RT	NM		NM	NM
Saif et al. [26]	2017	38	F	Abdominal pain	The body and tail of the pancreas	10	Distal pancreatectomy, splenectomy	Adjuvant VAC-IE	No		No	6 ANED
Komforti et al. [27]	2018	39	M	Abdominal and back pain, loss of appetite, loss of weight, dehydration	Body of the pancreas	8	Open laparotomy, surgical biopsies	NM	Yes	1	ERCP with sphincterotomy and stent placement, celiac plexus block, oral pain medication	NM
Liu et al. [28]	2018	36	M	Abdominal pain, jaundice	Head of the pancreas	6.3	Roux-en-Y choledochojejunostomy	Adjuvant CHT + RT	Yes	1	No	2 DOC
Pinheiro et al. [29]	2019	22	M	Abdominal pain, precocious gastric fullness, abdominal mass, weight loss	The body and tail of the pancreas	NM	Resection of body and tail of the pancreas, spleen, large omentum, gastric antrum, body (Roux-en-Y reconstruction); a small portion of hepatic segment III, gallbladder, dissection of the hepatic hilus with ligature of the splenic vein by the formation of the portal vein	Adjuvant VAC-IE	No	-	-	96 ANED
Achufusi et al. [30]	2020	13	F	Dyspepsia, exopthalmus	Head of the pancreas	NM	Removal of large areas of the tumor and the associated pancreatic pseudocyst; subsequent surgery was performed 3 days later with subtotal excision of a large pancreatic pseudocyst, cholecystectomy, distal gastrectomy, and Roux-en-Y gastrojejunostomy	No adjuvant treatment	No	-	-	DOD
Miller et al. [31]	2020	41	F	Jaundice, gray stools	Head of the pancreas	5.1	Scheduled for Whipple	Neoadjuvant CHT	NM	NM	NM	5 AWD
Miller et al. [31]	2020	39	F	Abdominal pain, jaundice	The body and tail of the pancreas	12.5	Scheduled for Whipple	Neoadjuvant CHT	NM	NM	NM	4 AWD
Miller et al. [31]	2020	78	F	Abdominal pain, jaundice	Head of the pancreas	3.2	Whipple	Unknown history of adjuvant therapy	NM	NM	NM	71 ANED
Miller et al. [31]	2020	27	M	Abdominal pain	Head of the pancreas	3	Whipple	Neoadjuvant CHT	NM	NM	NM	36 ANED
Miller et al. [31]	2020	20	M	Abdominal pain, jaundice	Head of the pancreas	3.5	Whipple	Adjuvant CHT	NM	NM	NM	111 DOD
Miller et al. [31]	2020	19	M	Abdominal pain	Body of the pancreas, retroperitoneal, and kidney extension	6	En bloc pancreas and kidney resection	Neoadjuvant CHT	NM	NM	NM	30 DOD
Miller et al. [31]	2020	22	M	Abdominal pain	Body of the pancreas, retroperitoneal extension	17	En bloc pancreas, spleen, and kidney resection	NM	NM	NM	NM	84 ANED
Miller et al. [31]	2020	17	M	Abdominal pain, jaundice	Head of the pancreas	9	Whipple	Adjuvant CHT	NM	NM	NM	120 ANED
Miller et al. [31]	2020	35	M	Shortness of breath, gastrointestinal bleeding, anemia	Peripancreatic soft tissue, 0.5 cm from the ampulla of Vater	6.5	Whipple	Adjuvant VAC-IE	NM	NM	NM	120 ANED
Miller et al. [31]	2020	15	M	Abdominal pain	Head of the pancreas	NM	Whipple	Adjuvant CHT	Yes	60	Resection + CHT	NM
Miller et al. [31]	2020	64	F	NM	Head of the pancreas	NM	Whipple	NM	NM	NM	NM	DOD
Miller et al. [31]	2020	33	M	NM	Head of the pancreas	NM	Whipple	NM	NM	NM	NM	NM
Miller et al. [31]	2020	23	M	NM	Head of the pancreas	6	Whipple	NM	NM	NM	NM	DOD
Yohannan et al. [32]	2020	26	F	Abdominal pain, nausea, vomiting	Body and tail of the pancreas, subdiaphragmatic nodule, posterior gastric wall invasion, 2 pericolic soft tissue lesions	10	Distal pancreatectomy, splenectomy, subtotal gastrectomy with Roux-en-Y gastrojejunostomy, left colectomy	Neoadjuvant vincristine, ifosfamide, and doxorubicin + adjuvant vincristine, temozolomide, and irinotecan	Yes	NM	Hospice care	DOD
Bakshi et al. [33]	2021	17	F	Yellowish discoloration of sclera, itching with loss of weight and appetite	Head and uncinate process of the pancreas	7.5	Whipple	NM	NM	NM	NM	NM
Liu et al. [34]	2022	16	M	Abdominal pain	Tail of the pancreas	9.3	Total mass excision, nephrectomy, partial adrenalectomy	Neoadjuvant vincristine, epirubicin, and cyclophosphamide–IE; adjuvant vincristine, epirubicin, and cyclophosphamide–IE + RT	NM	NM	NM	48 ANED
Singh et al. [36]	2023	18	F	Abdominal pain, bloating, nausea, abdominal mass	The body and tail of the pancreas	15.7	No	VAC	No	-	-	1 DOD
Gecici et al. [35]	2023	4	F	Vomiting	The body and neck of the pancreas	5	Central pancreatectomy, Roux-en-Y pancreaticojejunostomy	Adjuvant vincristine, doxorubicin, cyclophosphamide, ifosfamide, etoposide, and topotecan	No	-	-	12 ANED
Wang et al. [37]	2023	43	M	Abdominal pain	Body and tail of the pancreas, adrenal metastases	6.5	Radical surgery for pancreatic cancer and left adrenalectomy	Adjuvant VAC-IE	NM	NM	NM	4 ANED
Liu et al. [38]	2024	8	M	Palpable mass in the upper abdominal region	Head of the pancreas, transverse colon, and small intestine invasion	12	Whipple and partial transverse colectomy	Adjuvant VAC-IE	No	-	No	30 ANED
Fatima et al. [39].	2024	28	M	Abdominal pain, loss of appetite, loss of weight	The body and tail of the pancreas	20	Resection of the pancreatic mass, omentectomy, and splenectomy	No adjuvant treatment	NM	NM	NM	Lost to follow-up
Current case	2024	29	M	Abdominal pain, jaundice	Head of the pancreas	4.2	Whipple	VAC-IE	Yes	24		54 DOD

Abbreviations: ANED: alive with no evidence of disease; AWD: alive with disease; CHT: chemotherapy; DOC: died of other cause; DOD: died of disease; mo: months; NM: not mentioned; RT: radiotherapy; VAC: vincristine, adriamycin, cyclophosphamide; VAC-IE: vincristine, adriamycin, cyclophosphamide–ifosfamide, etoposide.

**Table 2:** Clinicopathological and treatment data of metastatic ESs to the pancreas.

Authors	Year	Age	Gender	Clinical Presentation	Site	Tumor Size (cm)	Surgery	Adjuvant/Neoadjuvant CHT/RT	Recurrence	Interval to Recurrence (Months)	Second-Line Therapy	Outcome
Pappo et al. [40]	1989	13	M	Headaches, papilledema, retinal hemorrhage, gingival hypertrophy	Bone marrow, peripancreatic and retroperitoneal lymph nodes, body and tail of the pancreas, right testicle, left humerus, left distal tibia, skull, sella turcica, dura, scalp, multiple bones, cervical lymph nodes, liver, spleen, lungs, diaphragm, psoas muscle, the left ventricular septum of the heart	NM	None	Prednisone, vincristine, and cyclophosphamide (for lymphoma diagnosis)	Yes	1	CHT	5 DOD
Mulligan et al. [41]	1997	26	M	Knee pain	Proximal end of the femur, body and tail of the pancreas	NM	Open surgical biopsy, fine-needle aspiration biopsy	Therapeutic CHT + RT	Yes	11	CHT	15 DOD
Shi et al. [42]	2013	19	M	Asymptomatic/abdominal pain	Lung (left lateral basal segment/head of the pancreas	5.5 (lung), 4 (pancreas)	Lower left lung lobectomy + lymph node dissection	No (patient refusal)	Yes	17	Whipple, no CHT (patient refusal)	84 AWD
Kapatia et al. [43]	2020	28	F	Abdominal pain, vomiting	Pubic bone, pancreas	3 (pancreas)	None	NM	Yes	12	NM	NM
Polimera et al. [44]	2020	29	M	Findings during follow-up	Triceps, lung, orbit, neck of the pancreas—3rd recurrence	2.1 (pancreas)	NM	VAC-IE + RT (main disease)	Yes (3)	168, 198	1st recurrence: limb-sparing resection/temozolomide/irinotecan × 2; cyclophosphamide and topotecan × 6; 2nd recurrence: 8 courses of cyclophosphamide, topotecan + RT	402 AWD
Guduguntla et al. [45]	2022	37	M	Nausea, vomiting, epigastric pain, urinary urgency, polyuria, dysuria	Pelvic mass, the body of the pancreas	5.1 (pelvic mass), 2.8 (pancreas)	None	Cisplatin and etoposide (SCLC diagnosis)/VAC + RT	No	Simultaneous	NA	12 AWD
Kapoor et al. [46]	2022	21	M	NM	Tail of the pancreas	NM	None	Vincristine, doxorubicin, ifosfamide + RT	Yes	36	Cyclophosphamide, topotecan; planned for surgery	40 AWD
Sarmast et al. [47]	2023	13	M	Epistaxis from left nostril, intermittent nasal blockage, rhinorrhea, pain in both legs, weight loss, loss of appetite	Left nostril, bilateral scapulae, multiple ribs, vertebral bodies, left anterior chest wall, head, uncinate process, tail of the pancreas	NM	None	VAC (vincristine, adriamycin, and cyclophosphamide) alternating with IE (ifosfamide and etoposide)	No	Simultaneous	NA	NM

Abbreviations: AWD: alive with disease; CHT: chemotherapy; DOD: died of disease; NM: not mentioned; RT: radiotherapy.

## Supplementary Materials

The following supporting information can be downloaded at https://www.mdpi.com/article/10.3390/diagnostics14232694/s1: Table S1: Comparisons between the patients with metastatic disease and primary sarcomas. Table S2: Comparisons between the patients with and without recurrence. Bold *p* values indicate statistical significance.
