# Supplementary material for: Primary and Metastatic Pancreatic Ewing Sarcomas: A Case Report and Review of the Literature
Source: Diagnostics (Basel). 2024 Nov 29;14(23):2694. doi: 10.3390/diagnostics14232694 (PMC11639833; doi:10.3390/diagnostics14232694)
Supplement: Supplementary file 1 [file diagnostics-14-02694-s001.zip › diagnostics-3262531-supplementary.pdf]

Table S1: Comparisons between the patients with metastatic disease and primary. Bold p values indicate statistical significance.

	Metastatic (N=8)	Primary (N=52)	P-value
**Age**			
Mean (SD)	23.3 (8.33)	26.1 (15.1)	0.802 kw
Median [Min, Max]	23.5 [13.0, 37.0]	22.5 [2.00, 78.0]	
**Gender**			
F	1 (12.5%)	22 (42.3%)	0.133 fx
M	7 (87.5%)	29 (55.8%)	
Missing	0 (0%)	1 (1.9%)	
**Symptom abdominal pain**			
No	5 (62.5%)	15 (28.8%)	0.0857 fx
Yes	2 (25.0%)	33 (63.5%)	
Missing	1 (12.5%)	4 (7.7%)	
**Symptom jaundice**			
No	7 (87.5%)	34 (65.4%)	0.172 fx
Yes	0 (0%)	14 (26.9%)	
Missing	1 (12.5%)	4 (7.7%)	
**Symptom other**			
No	2 (25.0%)	24 (46.2%)	0.426 fx
Yes	5 (62.5%)	24 (46.2%)	
Missing	1 (12.5%)	4 (7.7%)	
**Site Body**			
No	5 (62.5%)	36 (69.2%)	0.699 fx
Yes	3 (37.5%)	16 (30.8%)	
**Site Head**			
No	6 (75.0%)	21 (40.4%)	0.124 fx
Yes	2 (25.0%)	31 (59.6%)	
**Site Neck**			
No	7 (87.5%)	51 (98.1%)	0.251 fx
Yes	1 (12.5%)	1 (1.9%)	
**Site Tail**			
No	4 (50.0%)	38 (73.1%)	0.225 fx
Yes	4 (50.0%)	14 (26.9%)	
**Site Other**			
No	1 (12.5%)	40 (76.9%)	<0.001 fx
Yes	7 (87.5%)	12 (23.1%)	
**Tumor size (cm)**			
Mean (SD)	NA (NA)	8.04 (4.53)	NA
Median [Min, Max]	NA [NA, NA]	6.50 [3.00, 20.0]	
Missing	8 (100%)	8 (15.4%)	
**Recurrence**			
No	2 (25.0%)	11 (21.2%)	0.42 fx
Yes	6 (75.0%)	13 (25.0%)	
Missing	0 (0%)	28 (53.8%)	
**Interval to recurrence (months)**			
Mean (SD)	40.8 (63.4)	18.3 (19.5)	0.509 kw
Median [Min, Max]	14.5 [1.00, 168]	10.5 [1.00, 60.0]	
Missing	2 (25.0%)	40 (76.9%)	
**Survival (months)**			
Mean (SD)	93.0 (154)	33.8 (34.6)	0.439 kw
Median [Min, Max]	27.5 [5.00, 402]	28.5 [0, 120]	
Missing	2 (25.0%)	16 (30.8%)	
**Patient status**			
AWD	4 (50.0%)	6 (11.5%)	**0.0183 fx**

	Metastatic (N=8)	Primary (N=52)	P-value
DOD	2 (25.0%)	13 (25.0%)	
ANED	0 (0%)	18 (34.6%)	
DOC	0 (0%)	3 (5.8%)	
Missing	2 (25.0%)	12 (23.1%)	
**Life status**			
Alive	4 (50.0%)	27 (51.9%)	1 fx
Dead	2 (25.0%)	13 (25.0%)	
Missing	2 (25.0%)	12 (23.1%)	

AWD: Alive with disease, DOD: Died of Disease, ANED: Alive no evidence of disease, DOC: died of other cause, fx: Fisher exact test, kw: Kruskal Wallis test, NA: Not Applicable

Table S2: Comparisons between the patients with and without recurrence. Bold p values indicate statistical significance.

	No recurrence (N=13)	Recurrence (N=19)	P-value
**Group**			
Metastatic	2 (15.4%)	6 (31.6%)	0.42 fx
Primary	11 (84.6%)	13 (68.4%)	
**Age**			
Mean (SD)	22.1 (10.6)	23.5 (9.93)	0.7 tt
Median [Min, Max]	22.0 [4.00, 38.0]	26.0 [6.00, 39.0]	
**Gender**			
F	7 (53.8%)	5 (26.3%)	0.15 fx
M	6 (46.2%)	14 (73.7%)	
**Symptom abdominal pain**			
No	7 (53.8%)	6 (31.6%)	0.519 x2
Yes	6 (46.2%)	11 (57.9%)	
Missing	0 (0%)	2 (10.5%)	
**Symptom jaundice**			
No	12 (92.3%)	13 (68.4%)	0.355 fx
Yes	1 (7.7%)	4 (21.1%)	
Missing	0 (0%)	2 (10.5%)	
**Symptom other**			
No	2 (15.4%)	8 (42.1%)	0.119 fx
Yes	11 (84.6%)	9 (47.4%)	
Missing	0 (0%)	2 (10.5%)	
**Site Body**			
No	6 (46.2%)	14 (73.7%)	0.15 fx
Yes	7 (53.8%)	5 (26.3%)	
**Site Head**			
No	7 (53.8%)	11 (57.9%)	1 x2
Yes	6 (46.2%)	8 (42.1%)	
**Site Neck**			
No	12 (92.3%)	18 (94.7%)	1 fx
Yes	1 (7.7%)	1 (5.3%)	
**Site Tail**			
No	6 (46.2%)	12 (63.2%)	0.556 x2
Yes	7 (53.8%)	7 (36.8%)	
**Site Other**			
No	10 (76.9%)	10 (52.6%)	0.267 fx
Yes	3 (23.1%)	9 (47.4%)	
**Tumor size (cm)**			
Mean (SD)	11.2 (6.47)	7.45 (4.29)	0.176 kw
Median [Min, Max]	11.5 [3.00, 20.0]	6.15 [3.50, 18.0]	
Missing	4 (30.8%)	7 (36.8%)	
**Interval to recurrence (months)**			
Mean (SD)	NA (NA)	25.8 (39.3)	NA kw
Median [Min, Max]	NA [NA, NA]	12.0 [1.00, 168]	
Missing	13 (100%)	1 (5.3%)	
**Survival (months)**			
Mean (SD)	21.6 (28.5)	53.9 (98.8)	0.133 kw
Median [Min, Max]	12.0 [1.00, 96.0]	30.0 [2.00, 402]	
Missing	3 (23.1%)	4 (21.1%)	
**Patient status**			
ANED	7 (53.8%)	1 (5.3%)	**0.00725 fx**
AWD	1 (7.7%)	5 (26.3%)	

	No recurrence (N=13)	Recurrence (N=19)	P-value
DOC	1 (7.7%)	1 (5.3%)	
DOD	2 (15.4%)	9 (47.4%)	
Missing	2 (15.4%)	3 (15.8%)	
**Life status**			
Alive	9 (69.2%)	7 (36.8%)	0.109 fx
Dead	2 (15.4%)	9 (47.4%)	
Missing	2 (15.4%)	3 (15.8%)	

AWD: Alive with disease, DOD: Died of Disease, ANED: Alive no evidence of disease, DOC: died of other cause, fx: Fisher exact test, kw: Kruskal Wallis test, tt: t-test, x2: χ-square test, NA: Not Applicable
